# Comparison of the effectiveness of oral morphine versus oral tramadol on early pain control in opioid-naive patients with moderate cancer pain

**DOI:** 10.3332/ecancer.2025.1864

**Published:** 2025-03-05

**Authors:** Ramila Shilpakar, KC Anuj, Bibek Acharya, Sandhya Chapagain, Shama Pandey, Prakash Neupane, Bishal Poudel, Soniya Dulal, Bishnu Dutta D Poudel

**Affiliations:** 1 Department of Clinical Oncology, National Academy of Medical Sciences (NAMS), Bir Hospital, 44600, Kathmandu, Nepal; 2 The University of Kansas Medical Center, 2330 Shawnee Mission Parkway, Westwood, Kansas City, KS 66205, USA; 3 Institute of Medicine, Tribhuvan University Teaching Hospital, 44600, Kathmandu, Nepal; 4 BP Koirala Institute of Health Sciences, 56705, Dharan, Nepal; 5 KIST Medical College, 44700, Lalitpur, Nepal

**Keywords:** moderate cancer pain, morphine, tramadol

## Abstract

**Purpose:**

The purpose of this study was to compare the efficacy of oral morphine (MOR) with oral tramadol (TRM) in control of pain as well as physical well-being in patients (pts) with moderate cancer pain (MCP) using the Edmonton Symptom Assessment Scale (ESAS).

**Methods:**

An Institutional Review Board (IRB) approved randomised phase II trial was performed in opioid-naive pts with MCP as defined by pain score in numerical rating score (NRS) of 4–6. Patients were randomised to receive MOR syrup 5 mg 4 hourly or TRM 50 mg four times a day. Titration of dose was done in both groups for 3 days in case of inadequate pain control as per standard recommendation for MOR or until the maximum recommended daily dose for TRM. MOR was changed to prolonged release form on Day 4. The primary endpoint was the number of early responders, defined as pts with at least 20% reduction in pain intensity on NRS on Day 3. The secondary outcome was the number of patients with highly meaningful pain reduction, defined as a decrease in pain intensity on NRS by ≥5 and improvement in physical well-being with ESAS at Day 7.

**Results:**

Sixty-eight pts consented and were randomised, 34 in each arm. The primary endpoint occurred in 94.1% pts in MOR and 55.9% in TRM (*p* < 0.001). The number of patients with highly meaningful pain reduction was significantly higher in MOR than in TRM (76.5% versus 32.35%; *p* < 0.001). Improvement in general physical well-being as assessed by ESAS was better in the MOR group. No difference in adverse effects was noted between the treatment arms.

**Conclusion:**

In this study, MOR was superior to TRM in the control of pain with statistically significant differences in the primary and secondary endpoints. Therefore, early use of MOR skipping the World Health Organization sequential analgesic ladder for MCP may be a higher value option in resource-scarce country with limited access to healthcare.

## Introduction

Pain is one of the most common symptoms associated with cancer with higher prevalence and greater intensity reported with more advanced disease stages [1]. Unrelieved pain causes patient discomfort and greatly affects their activities, motivation, interactions with family and friends and overall quality of life [2, 3]. The quality of life in cancer patients can be improved by good symptom control even when the disease is incurable [4].

Different types of pain occur in patients with cancer. Failure to adequately assess pain frequently leads to poor pain management. A comprehensive evaluation to find the cause of the pain and identify optimal therapies is essential to ensure proper pain management [5]. Studies have shown show relatively high prevalence of undertreated cancer pain in Asia, the weighted mean being 45.2% in Asia compared with 20.2%, 29.5% and 32.0% in Australia, Europe and North America, respectively [6]. The estimated adequacy of treatment for cancer pain identified that approximately 32% of patients were not receiving analgesia proportionate to their pain severity and a significant number of patients with cancer pain are not well managed [7]. Socioeconomic burden associated with cancer exacerbates the consequences of inadequate pain management in addition to other barriers such as inadequate assessment of pain, opioid access and regulations and stigmas associated with opioid use [8].

Multiple professional oncology bodies have published pain management guidelines addressing knowledge gaps in order to help healthcare providers effectively manage cancer pain [9–13]. The leading principle for pain management of cancer pain today is the World Health Organization (WHO) pain ladder. Recommendations include the use of quantitative pain assessment tools and a step-up prescription of analgesics in the order of nonopioids (step I), weak opioids (step II) and strong opioids (step III) until adequate relief from pain is achieved [14]. Unrelieved pain remains to be a substantial concern in patients with cancer despite the widespread use of WHO analgesic ladder [6, 15, 16]. The usefulness of sequential WHO analgesic ladder, in particular step II opioids, is becoming debatable. The use of strong opioids instead when NSAIDs become ineffective is being forwarded as studies have shown that the balanced use of opioids especially morphine (MOR) is much more effective in relieving pain [8, 17–19].

Nepal is low middle income country (LMIC) with a high cancer burden and significant palliative care needs, especially adequate pain control [20]. With the improved availability of opioids in Nepal in resource-limited setting, where the cost effectivity of any treatment is a major issue, the two-step approach of nonopioids and strong opioids may be more practical [21, 22]. Therefore, the use of MOR for moderate cancer pain (MCP) rather than sequential WHO analgesic ladder with tramadol (TRM) seems reasonable in the setting of limited access to healthcare for early and effective pain control.

## Patients and methods

This was a randomised phase II study conducted at the Department of Clinical Oncology, National Academy of Medical Sciences (NAMS), Bir Hospital, Kathmandu, Nepal. Approval was obtained from the IRB, NAMS, Bir Hospital, and informed written consent was obtained from each participant.

The study included opioid-naive cancer patients or patients with no opioid use in the past 30 days with MCP (4–6 on the standard numerical rating scale (NRS) [23], range 0–10), aged ≥18 years who gave informed written consent. Patients with the ability to swallow oral medication and ECOG PS 0–4 were included. Exclusion criteria were: deranged renal function (creatinine clearance < 20 mL/minute); deranged liver function defined as serum bilirubin >5 times upper limit and/or alanine transaminase/aspartate transaminase >5 times upper limit; history of adverse reactions or allergy to any of the study medications; patients who were receiving a course of radiotherapy or radioiodine therapy to obtain pain relief or for whom such treatment was completed less than 14 days or was planned within 1 week; evidence of respiratory depression with resting respiratory rate of less than 8/minute; uncontrolled nausea, vomiting or evidence of gastrointestinal tract obstruction; and pregnant or breast feeding.

Randomization was 1:1 using a simple randomization method. Sixty-eight envelopes were marked either arm A or arm B and patients blindly chose one envelope.

### Study treatment and assessment

Patients were assigned into two groups. The MOR group received oral normal-release morphine (NRM) at a starting dose of 5 mg every 4 hours. A double dose (10 mg) was administered at bedtime to avoid nocturnal dosing. The duration of the titration phase was 3 days. Patients who did not experience satisfactory pain relief during the interval between one dose and the next could took rescue doses of oral NRM, up to a maximum of one dose every hour; rescue NRM doses were the same as the patient’s regular doses. The dosage was retitrated on a daily basis so that the dosage of oral NRM to be given in the next 24 hours was based on the total opioid dose (regular plus rescue) taken by the patient. Outpatients had ambulatory visits during the titration phase if feasible, which was substituted from Day 2 by phone calls to monitor the intensity of pain, the dosage of the drug and the onset of other symptoms if not feasible.

The TRM group received a normal-release formulation of TRM 50-mg PO QID for 72 hours. Monitoring of side effects was done on Days 3 and 7. Titration was done for 3 days. If there is no improvement in pain on Day 2 or 3, the dose of TRM was increased to the maximum recommended dose of 400 mg for adults and 300 mg for patients aged >75 years.

In both groups, patients were evaluated on 4th day for pain control. In both groups, during the study, it was allowed to: give the maximum recommended dose for paracetamol of 4,000 mg/day to patients in both arms. Any need for other adjuvants (steroid, antidepressant and anticonvulsant) for pain control was considered in both groups. Increase or decrease the dosage of assigned treatment to obtain adequate pain control with acceptable side effects. After the titration phase, in case of breakthrough pain, extra doses of MOR, i.e., 1/6 of the daily dose in the MOR group and extra doses of the TRM up to the maximum daily dose, were given in both groups.

Patients were monitored on Days 3 and 7 after randomization.

### Study endpoints

The primary endpoint was the number of early responders, defined as pts with at least 20% reduction in pain intensity on NRS on Day 3. The secondary outcome was the number of patients with highly meaningful pain reduction, defined as a decrease in pain intensity on NRS by ≥5 and improvement in physical well-being with the Edmonton Symptom Assessment Scale (ESAS) on Day 7.

### Data collection and statistical analysis

Data collection was done on a standardised data collection sheet.

The sample size was calculated based on a confidence level of 95%, a confidence interval of 10% and a statistical power of 80%. The calculated sample size was 56%.

Considering a dropout of about 25% of patients, in order to obtain the required number of patients, 68 patients were enrolled. Analysis of data was performed upon completion of the study. The data were entered using SPSS software. Statistical analysis was also done using SPSS software after entering the data on a master chart. Data were analysed using descriptive statistical methods such as mean and standard deviation. Variables were correlated with each other and tested for statistical significance using the chi-square test. A 95% confidence interval and *p*-value less than 0.05 were termed as statistically significant.

## Results

Sixty-eight pts consented and were randomised, 34 in each arm.

The baseline characteristics of the patients in the study were shown in Table 1. The median ages were similar in both groups, 56 years in the MOR and 53 years in the TRM group. Characteristics of pain according to the study group have been shown in Table 2. The majority of patients had somatic and mixed types of pain at randomization.

The mean baseline pain score was also similar in both groups: 5.4 ± 0.7 in the MOR group and 5.3 ± 0.6 in the TRM group (*p* = 0.2). The baseline mean ESAS scores were also similar in both groups 28.8 and 27.2 in the MOR and TRM groups, respectively, which was statistically not significant.

**Figure 1.**
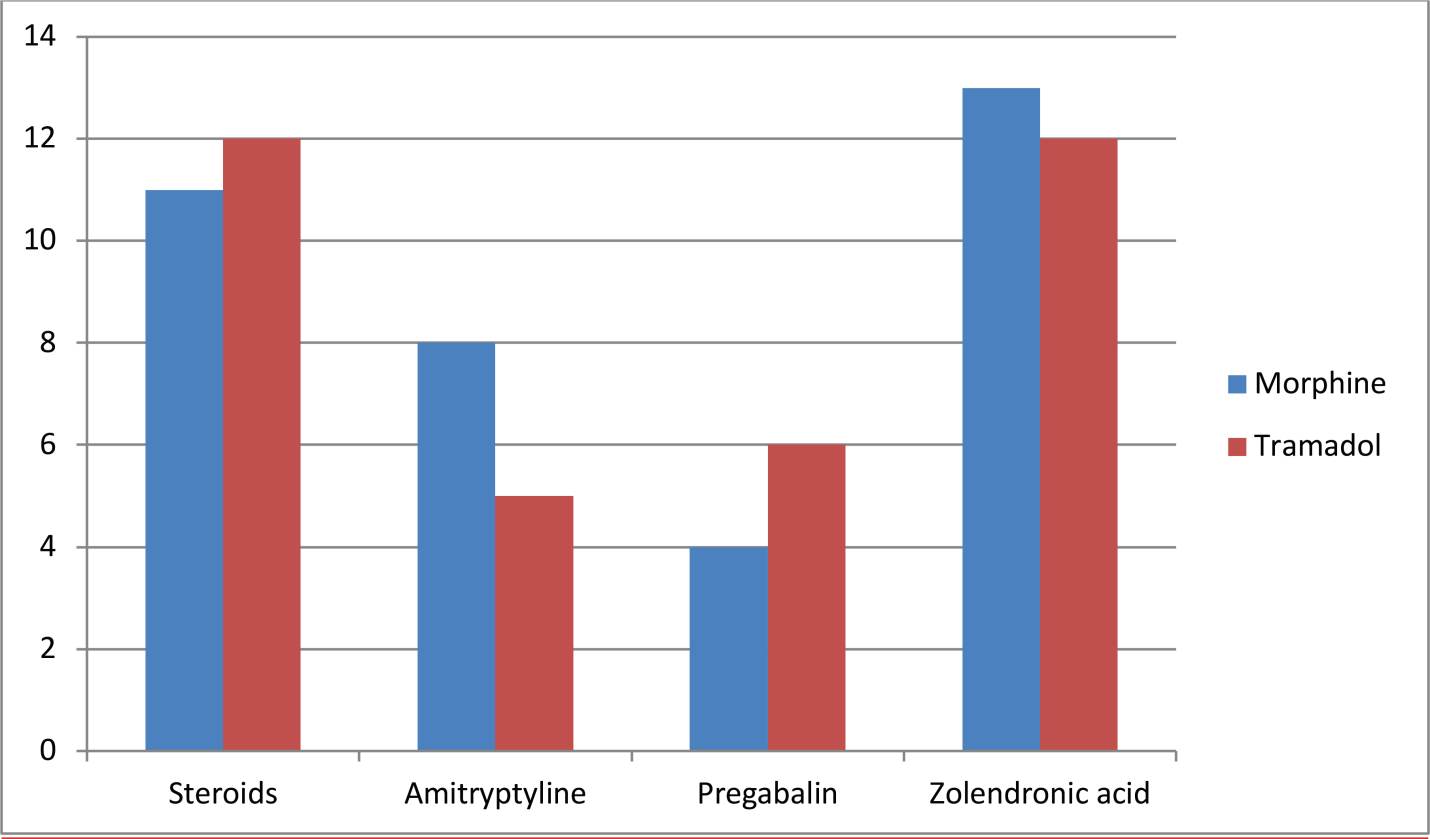
Different adjuvants used in the study group.

The primary endpoint occurred in 94.1% pts in MOR and 55.9% in TRM (*p* < 0.001) as shown in Table 3. The number of patients with highly meaningful pain reduction was significantly higher in MOR than in TRM (76.5% versus 32.35%; *p* < 0.001) (Table 4). Improvement in general physical well-being as assessed by ESAS was better in the MOR group. The mean ESAS score at Day 7 was 12.4 in the MOR group compared with 15.6 in the TRM group, which is statistically significant (*p* = 0.01) as shown in Table 5. Subset analysis showed shortness of breath was better in the MOR group compared with the TRM group. Adjuvants were used in both groups. Of the total study population, 53% of patients had some form of adjuvant treatment; 50% of the patients in the MOR group and 56% of the patients in the TRM group had adjuvants used as shown in Figure 1. The adjuvants used were similar in both the study groups. Side effects according to the study group are shown in Table 6. Though statistically not significant, constipation was more common in the MOR group, while dizziness was seen more in the TRM group.

## Discussion

Adequate pain control, an essential part of cancer care still remains a challenge in resource-limited countries like Nepal. Early use of MOR for MCP rather than sequential WHO analgesic ladder seems reasonable in the setting of limited access to healthcare. The purpose of this study was to compare the efficacy of oral MOR with oral TRM in control of pain as well as physical well-being in patients with MCP using the Edmonton Symptom Assessment Scale (ESAS).

In this study, regarding the primary endpoint of assessing the efficacy of MOR versus TRM, the numbers of early responders were higher in the MOR compared with the TRM group. The number of early responders, defined as patients with at least 20% reduction in pain intensity on NRS on Day 3, was statistically significant in the MOR group 32 (94.1%) compared with the TRM group 19 (55.9%). The advantage of MOR over TRM was already evident after 3 days and remained constant at each follow-up. These findings are comparable with a study by Bandieri *et al* [24], in which the primary endpoint of pain reduction of 20% or more from baseline was achieved in 88.2% of patients (97 of 110) in the MOR group and 54.7% of patients (64 of 117) in the weak opioids group (*p* < 001).

The change in mean ESAS score was also very significant in the MOR group compared with the TRM group. Therefore, MOR was more effective in improving general physical well-being than TRM in patients with MCP. Subset analysis of ESAS score revealed an improved score in the shortness of breath domain of ESAS score in the MOR group in comparison to the TRM group. These findings advocate further argument in favour of its use in opioid-naive patients with cancer with moderate pain.

We acknowledge that a limitation of this study was its small sample size. However, given these initial positive results and the possibility of significant improvement in pain control and improved quality of life, larger randomised trials seem warranted as value-based care is important in all countries. Another weak opioid like codeine was not compared in this study and longer follow-up was not done to see side effects and outcomes in a longer run. The follow-up period was only 7 days, which is short to evaluate the overall efficacy of the treatment in the long term that may represent a bias in the interpretation of the result. Long-term follow-up studies are essential to interpret the results.

Based on this study, MOR was superior to TRM in the control of pain with statistically significant differences in the primary and secondary endpoints. Therefore, early use of MOR skipping the WHO sequential analgesic ladder for MCP may be a higher value option in resource-scarce country with limited access to healthcare.

## Conflicts of interest

All the authors have no conflicts of interest.

## Funding

The study was funded by Nepal Health Research Council Research Grant.

## Figures and Tables

**Table 1.** Baseline demographic and clinical characteristics of the study patients.

Characteristics	MOR (*N* = 34)	TRM (*N* = 34)	Total (*N* = 68)
Age (years) Median	56	53	55
Age (years) Range	18–86	22–74	18–86
Gender – *n* (%)			
Male	20 (58)	22 (65)	42 (62)
Primary site– *n* (%)			
Lung	10 (29)	9 (27)	19 (28)
Breast	6 (18)	7 (21)	13 (19)
GI tract	4 (12)	5 (15)	9 (13)
Sarcoma	3 (9)	4 (11)	7 (10)
Hepatobiliary	3 (9)	2 (6)	5 (7)
Haematological	4 (11)	5 (14)	9 (13)
Genitourinary	2 (6)	1 (3)	3 (5)
Others	2 (6)	1 (3)	3 (5)
ECGO PS–*n* (%)			
ECOG 1	11 (33)	10 (29)	21 (31)
ECOG 2	17 (50)	19 (56)	36 (53)
ECOG 3	6 (17)	5 (15)	11 (16)
Pain score(Mean)	5.4	5.3	(*p* = 0.2)

**Table 2.** Types of pain according to the study group.

Type of pain	MOR – *n* (%)	TRM – *n* (%)	Total – *n* (%)
Somatic	11 (33)	10 (29)	21 (31)
Visceral	7 (19)	9 (26)	16 (24)
Neuropathic	1 (3)	1 (3)	2 (3)
Somatic and visceral	11 (33)	8 (24)	19 (28)
Visceral and neuropathic	2 (6)	4 (12)	6 (8)
Somatic and neuropathic	1 (3)	1 (3)	2 (3)
Multiple mixed	1 (3)	1 (3)	2 (3)
Total	34	34	68

**Table 3.** Primary endpoint according to the study group.

MOR – *N* (%)		TRM – *N* (%)		*p*-value
**Early response**	**No early response**	**Early response**	**No early response**	<0.01
32 (94.1%)	2 (5.9%)	19 (55.9%)	15 (44.1%)	

**Table 4.** Secondary endpoint according to the study group: highly meaningful pain reduction.

MOR *N* (%)		TRM *N* (%)		*p*-value
**Highly meaningful pain reduction**	**No – highly meaningful pain reduction**	**Highly meaningful pain reduction**	**No – highly meaningful pain reduction**	<0.01
26 (76.5%)	8 (23.5%)	11 (32.4%)	23 (67.6%)	

**Table 5.** Secondary endpoint according to the study group: ESAS at Day 7.

Characteristics	MOR	TRM	*p*-value
ESAS score baseline (Mean ± SD)	28.8 ± 6.1	27.2 ± 5.0	0.1
ESAS score Day 7 (Mean ± SD)	12.4 ± 3.8	15.6 ± 5.2	0.01

**Table 6.** Side effects according to the study group.

Side effects	Group		Total – *n* (%)
	MOR – *n* (%)	TRM – *n* (%)	
Constipation	11 (32)	7 (20)	18 (28)
Nausea	7 (20)	10 (29.4)	17 (25)
Vomiting	1 (2.9)	4 (11.8)	5 (7.4)
Dizziness	4 (11.8)	7 (20)	11 (16.2)
Dry mouth	1 (2.9)	0 (0)	1 (1.5)
Drowsiness	1 (2.9)	0 (0)	1 (1.5)
None	20 (58.9)	22 (64.7)	42 (61.8)
Total	34	34	68
